# Evaluation of the Low Carb Program Digital Intervention for the Self-Management of Type 2 Diabetes and Prediabetes in an NHS England General Practice: Single-Arm Prospective Study

**DOI:** 10.2196/25751

**Published:** 2021-09-09

**Authors:** Charlotte Summers, Simon Tobin, David Unwin

**Affiliations:** 1 DDM Health Coventry United Kingdom; 2 Norwood Surgery Southport United Kingdom

**Keywords:** Low Carb Program, low carbohydrate, diabetes, type 2 diabetes intervention, diabetes prevention, self-management, behavior change, prediabetes

## Abstract

**Background:**

Type 2 diabetes mellitus has serious health consequences, including blindness, amputation, and stroke. Researchers and clinicians are increasingly in agreement that type 2 diabetes may be effectively treated with a carbohydrate-reduced diet. Digital apps are increasingly used as an adjunct to traditional health care provisions to support remote self-management of long-term health conditions.

**Objective:**

Our objective was to evaluate the real-world 12-month outcomes of patients prescribed the Low Carb Program digital health intervention at a primary care National Health Service (NHS) site. The Low Carb Program is a nutritionally focused, 12-session, digitally delivered, educational behavior change intervention for glycemic control and weight loss for adults with prediabetes and type 2 diabetes. The program educates and supports sustainable dietary changes focused on carbohydrate restriction by utilizing behavior change techniques, including goal setting, peer support, and behavioral self-monitoring, as well as personalized downloadable resources, including recipes and meal plans tailored to ethnicity, weekly shopping budget, and dietary preferences.

**Methods:**

This study evaluated the real-world outcomes of patients recruited to the Low Carb Program at an NHS general practice in Southport, United Kingdom. All of the NHS patients recruited to the program were diagnosed with type 2 diabetes or prediabetes and were given access to the program at no cost. A total of 45 participants, with a mean age of 54.8 years (SD 13.2), were included in the study. Women made up 42% (19/45) of the sample. The mean hemoglobin A_1c_ (HbA_1c_) of the sample was 56.7 mmol/mol (SD 16.95) and the mean body weight was 89.4 kg (SD 13.8).

**Results:**

Of the 45 study participants recruited to the program, all of them (100%) activated their accounts and 37 (82%) individuals reported outcomes at 12 months. All 45 (100%) patients completed at least 40% of the lessons and 32 (71%) individuals completed more than nine out of 12 core lessons of the program. Glycemic control and weight loss improved, particularly for participants who completed more than nine core lessons in the program over 12 months. The mean HbA_1c_ went from 58.8 mmol/mol at baseline to 54.0 mmol/mol, representing a mean reduction of 4.78 mmol/mol (SD 4.60; *t*_31_=5.87; *P*<.001). Results showed an average total body weight reduction of 4.17%, with an average weight reduction of 3.85 kg (SD 2.49; *t*_31_=9.27; *P*<.001) at the 12-month follow-up point.

**Conclusions:**

A digital app prescribed to adults with type 2 diabetes and prediabetes in a primary care setting supporting a transition to a low-carbohydrate diet has been shown to be effective in improving glycemic control and enabling weight loss. Further research to understand more about factors affecting engagement with the app and further positive health implications would be valuable.

## Introduction

### Background

Type 2 diabetes is a costly, chronic noncommunicable disease expected to affect 552 million people globally by 2030 [1]. The National Health Service (NHS) spends around £8.8 billion (US $11.1 billion) annually on the treatment of type 2 diabetes, with 80% spent on complications [2]. Globally, the burden of type 2 diabetes is estimated to exceed US $1.3 trillion [3]. In the developed world, individuals living with diabetes are managed by primary care teams, with medical consultation visits averaging less than 3 hours a year. Individuals are essentially on their own most of the time [4]. Because of this enormous gap between appointments, diabetes care is primarily dependent on personal self-management, which, if not performed, increases the risk of premature death, blindness, amputation, and kidney failure [5]. In reality, type 2 diabetes self-management is neither easy nor simple and requires time as well as numeracy and literacy skills [6].

As with many noncommunicable diseases, lifestyle is one of the main causes of prediabetes and type 2 diabetes, and improvements in parameters such as dietary composition, physical activity, and sedentary lifestyle are determinants for reducing the frequency of this type of pathology. Obesity is considered to be the cause of up to 80% of type 2 diabetes cases [7].

Losing weight can provide significant health benefits and losing excess body weight contributes to reduction in the risk of type 2 diabetes, heart disease, osteoarthritis, and sleep apnea [8]. In addition, the maintenance of good blood glucose control has benefits to patients, with every 1% (6.2 mmol/mol) reduction in hemoglobin A_1c_ (HbA_1c_) contributing to a 43% reduction in the risk of amputation, 14% reduction in risk of myocardial infarction, and 37% reduction in risk of microvascular complications [9]. Poor diabetes control is also associated with a higher risk of COVID-19 complications [10].

The benefits of a low-carbohydrate diet (<130 g of carbohydrate per day) on weight and type 2 diabetes management are increasingly recognized. Recent meta-analyses comparing the effects of low-carbohydrate and low-fat diets found a significantly greater reduction in body weight for the low-carbohydrate group [11,12]. Several low-carbohydrate randomized controlled trials focusing on people with type 2 diabetes mellitus reported similar findings, with significant reductions in weight and BMI at 6 months [12].

Systematic reviews of low-carbohydrate diets (defined as <130 g of carbohydrate per day) and very low–carbohydrate (or ketogenic) diets (defined as <30 g of carbohydrate per day) in obesity generally show either *no superiority* (ie, the low-carbohydrate diet had the same impact on weight and other markers as other diets, such as low-fat and calorie-controlled diets) or a *benefit* compared to other diets [13]. A meta-analysis found that low- and very low–carbohydrate diets led to greater weight loss than following a low-fat diet and concluded that a very low–carbohydrate diet may be an alternative tool that can used against obesity [14]. A recent systematic review and meta-analysis of published and unpublished randomized trial data evaluating low-carbohydrate diets (<130 g/day or <26% of a 2000 kcal/day diet) and very low–carbohydrate diets (<10% calories from carbohydrates) for at least 12 weeks in adults with type 2 diabetes found that on the basis of moderate- to low-certainty evidence, patients adhering to a low-carbohydrate diet for 6 months may experience remission of type 2 diabetes without adverse consequences [15].

Integrating digital technology into primary care can increase access to care, improve patient outcomes, and decrease costs. Digital technology, including smartphone apps, has the potential to augment and extend the reach of health services through self-management support impacting lifestyle behaviors [16]. Use of smartphone apps has been demonstrated to improve glycemic outcomes in people with type 1 and type 2 diabetes [17]. Although there is evidence to the contrary, of the 23 studies analyzed in the systematic review published by Schoeppe et al on the efficacy of apps in improving lifestyle, smartphones were only seen to have a favorable impact on food habits in five studies and resulted in increased physical activity in nine studies [18]. Even though recent systematic reviews have concluded that internet and mobile interventions can improve lifestyle behaviors, most studies had no more than 3 to 6 months of follow-up, which emphasizes the need for research in long-term interventions [19].

Researchers and clinicians are increasingly in agreement that type 2 diabetes may be effectively treated with a carbohydrate-reduced diet [20,21]. Interventions providing low-carbohydrate or very low–carbohydrate programs have been clinically demonstrated to support improvements in weight, blood glucose, and demedication [22-26]. Long-term studies of low-carbohydrate dietary approaches to treat type 2 diabetes and obesity, however, are limited, particularly among those that are delivered and supported remotely [23,24].

### The Low Carb Program

The Low Carb Program is a digitally delivered, structured, digital health intervention for adults with type 2 diabetes, prediabetes, and obesity. The app, which is NHS Apps Library–approved, is available on the web, mobile devices, smart watches, smart speakers, and smart assistants [26,27].

User data are used to personalize the experience that members receive, to improve patient engagement through individualization of the participant’s experience [28]. During registration, patients are instructed to select a health goal and input their current health status and demographics, including age, gender, ethnicity, and dietary preferences, all of which are used to personalize the participant’s experience of the platform. Participants are given access to therapeutic nutrition education modules. Education is personalized to the user’s health status, age, ethnicity, and dietary preferences. A new module is available each week over the course of 12 weeks. Lessons are taught through videos, written content, or podcasts of varying lengths (approximately 3 to 12 minutes long). The modules are designed to help participants gradually reduce their total carbohydrate intake to less than 130 grams per day. Much of the content of the Low Carb Program is focused on the reduction of processed and ultraprocessed foods as well as foods that are high in sugar and refined carbohydrates. The program supports users to sustainably replace starchy foods, such as potatoes or rice, with green leafy vegetables, healthy fats, and some protein. Participants are encouraged to select foods that are minimally processed, and the program emphasizes home cooking and food preparation. The program syllabus is provided in Table 1 and screenshots of the program are provided in Multimedia Appendix 1.

**Table 1:** Core syllabus of the Low Carb Program.

Lesson no.	Topic	Objective
1	Welcome to the type 2 diabetes program	Safety notes and alerts to medications that require the health care professional team’s assistance Benefits of a reduced carbohydrate diet for people with type 2 diabetes Welcome from Dr David Unwin and reference to patient’s golden opportunity for change
2	Type 2 diabetes and diet	Factors that affect blood glucose levels Encouragement to engage with their health care providers
3	Controlling portion sizes	Introducing visual methods for interpreting portion size
4	Real vs processed foods	Identifying and eliminating refined and processed food
5	Healthy and unhealthy fats	Discussion of fat types and making appropriate choices depending on goals
6	Vegetables	Demonstrating the carbohydrate content of vegetables and cooking methods
7	Fruit	Reviewing the amount of sugar and starch in fruits and vegetables
8	Snacks and desserts	Examining low-carbohydrate snack, dessert, and drink options
9	Drinks	Tips on alcohol and eating-out options
10	Eating out and takeaways	Managing eating on the go and when traveling Making healthier takeaway and food choices
11	Practical ways to eat fewer carbohydrates	Practical tips for reducing carbohydrate intake further Safety information—highlighting medications that require health care practitioner assistance
12	Intermittent fasting	Introducing the principles of reducing the eating window using the 16:8 model

The program, which is NHS-approved, encourages participants to make behavior changes based on “action points” or behavior change goals at the end of each education module, based loosely on Dr Unwin’s own in-clinic program [29].

In the Low Carb Program tailored for individuals with type 2 diabetes, the first 2 weeks of the program contain an explanation of the physiology of type 2 diabetes and the role of diet, including a description of how a low-carbohydrate diet can help manage postprandial blood glucose levels and weight. The subsequent modules explore strategies to reduce dietary sources of sugar, in particular high-starch foods, such as bread, pasta, and rice. Participants are encouraged to make portion control and carbohydrate-restriction decisions based on visual plate representations. In place of carbohydrate-rich foods, an increased intake of green vegetables, low–glycemic index fruits (eg, blueberries, strawberries, and raspberries), and fats (eg, from olive oil, butter, eggs, nuts, and full-fat dairy) are advocated. The program stresses the importance of regular contact with the participants’ health care providers for adjustments in medications in weeks 1, 2, and 12. Weeks 11 and 12, which concentrate on sustaining a lower-carbohydrate lifestyle, were co-designed with clinicians and patients after collecting feedback from 5000 patient users of the Low Carb Program.

Participants’ health goals are supported with behavior change resources that are available to download, including information sheets, meal plans, a recipe library, and suggested food substitution ideas, all tailored to the user’s preferences. Users are matched within the platform to a digital buddy and are given access to a peer-support forum available 24 hours a day. The platform also includes digital tools for submitting self-monitoring data on a number of different variables, including blood glucose levels, blood pressure, mood, sleep, food intake, and body weight. Participants can self-report and connect wearables to the platform. Previous research has found that these self-reported health outcomes can be quite close to data within medical records [21,22]. Behavior change is maintained through continual engagement, new modules, and nudges to track health outcomes and interact with the support community. Automated feedback and nudges are provided to users, based on their use of the program, through emails and native in-app push notifications, and participants are notified when the next week’s module is available. Examples of personalized patient journeys in the Low Carb Program are shown in Multimedia Appendix 2. The platform requests that users check in on their weight and HbA_1c_ goals at regular intervals set by the user, defaulted to 12 months, to ensure that users feel in control of their learning at their own pace. Family members and carers can sign up on behalf of vulnerable or elderly patients, share credential-based access to the platform, and impute data on the patient’s behalf.

**Figure 1:**
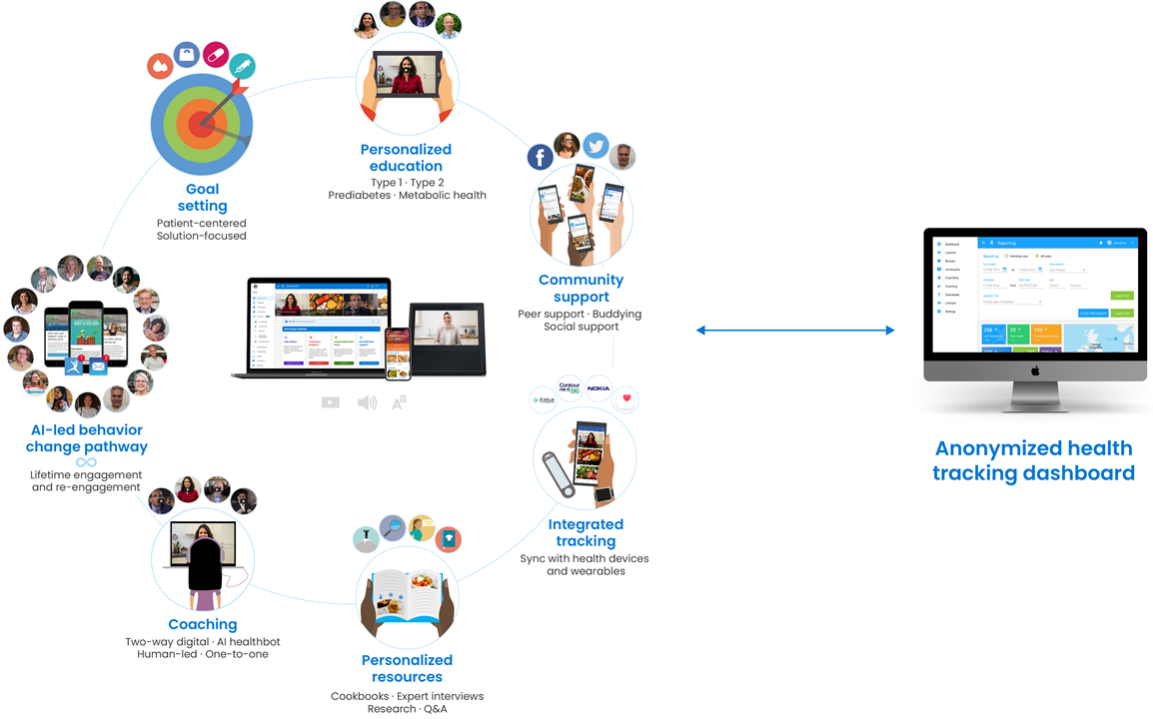
Architecture of the Low Carb Program digital health platform. AI: artificial intelligence.

The key elements that make up the Low Carb Program are grounded in the COM-B (Capability-Opportunity-Motivation Behavior) model of behavior change; the elements implement evidence-based behavior change techniques that are shown to be effective in digital platforms for behavior change interventions that support weight loss, increase physical activity, and improve self-efficacy of chronic disease management [28]. The platform was designed in full compliance with the NICE (National Institute for Health and Care Excellence) guideline NG183 [30]. See Figure 1 for an overview of the Low Carb Program architecture.

The platform has demonstrated clinical outcomes in patients with type 2 diabetes. Of 1000 patients followed for a year, participants with type 2 diabetes who completed the program reported an average of 7% loss in body weight and 1.2% or 13 mmol/mol HbA_1c_ reduction; in addition, 54% of patients eliminated or reduced medication. A total of 26% of patients who completed the program reported being in type 2 diabetes remission at 1 year [26].

### Objectives

This real-world study was conducted to evaluate the effectiveness of the digitally delivered Low Carb Program intervention at 12 months on the maintenance of glycemic control for NHS-recruited patients at Norwood Surgery in Southport, United Kingdom. We hypothesized that the use of the Low Carb Program would support the following improvements: better glycemic control, as measured by HbA_1c_, and weight loss.

## Methods

### Research Design

We used a single-arm pre-post intervention study design. Participants were not paid for their participation and were given access to the program for free. Participants provided informed consent regarding their anonymized data being used for analysis and publication.

### Participant Recruitment

**Figure 2:**
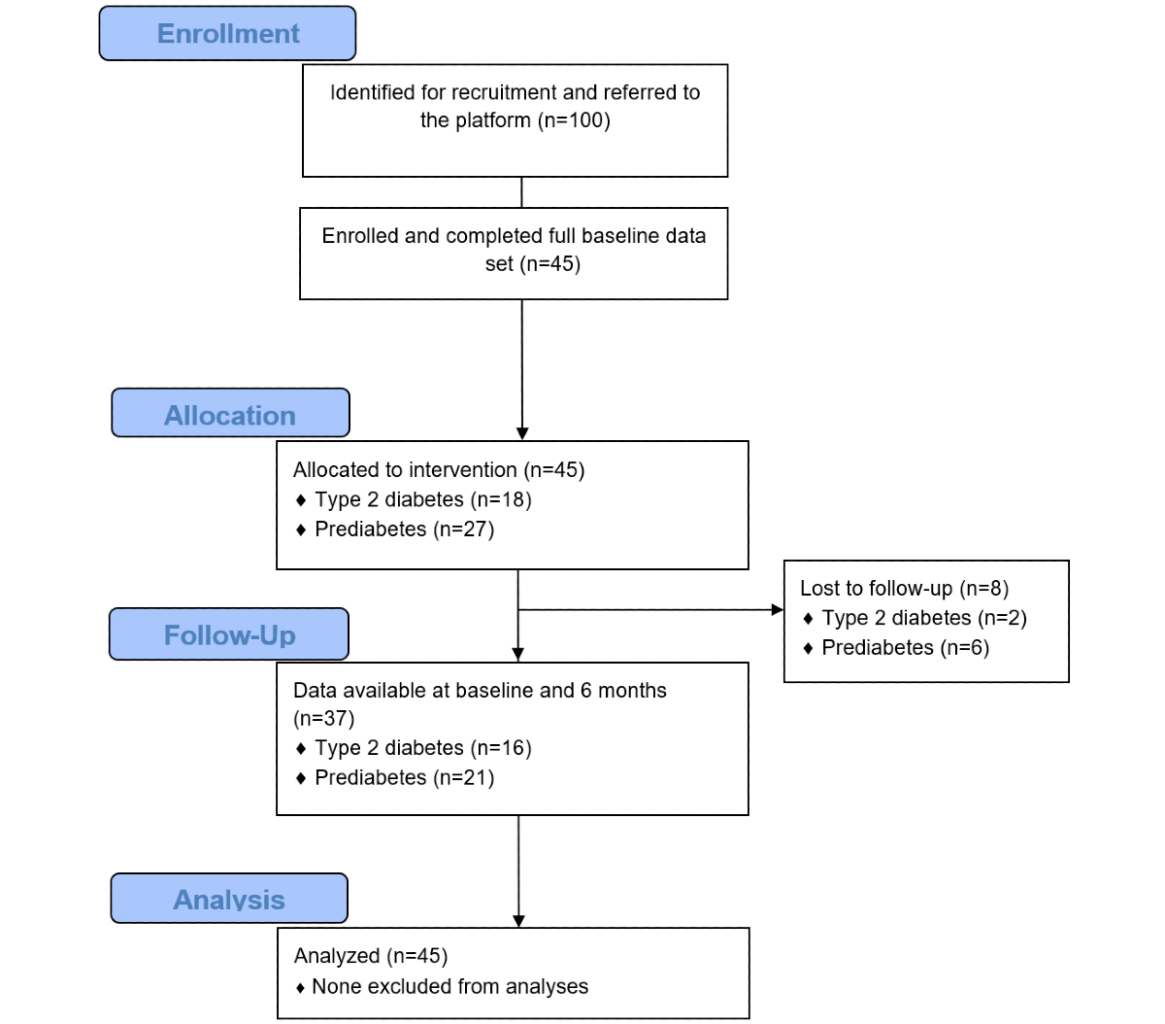
CONSORT (Consolidated Standards of Reporting Trials) diagram for participant inclusion in the study.

Participants were recruited from an NHS primary care setting—Norwood Surgery in Southport, United Kingdom—between April 19, 2018, and August 19, 2019. Patients aged 18 years or older with a confirmed diagnosis of type 2 diabetes or prediabetes who presented for any reason during the recruitment window were eligible for signposting if the consulting health care professional felt it was appropriate. See Figure 2 for a CONSORT (Consolidated Standards of Reporting Trials) flow diagram.

Patients who accepted signposting were given a Low Carb Program referral card, which was redeemed on the app or website. To have a broad applicability to a nonclinical trial setting, the only de facto exclusion criterion was the inability to understand English. A total of 100 referral cards were provided to the NHS general practice in Southport. A total of 45 participants signed up and all were followed for 12 months. The characteristics of the 55 participants who declined the referral card were not recorded.

### Measures

At baseline, participants recruited to the Low Carb Program input their type of diabetes, year of diagnosis, most recent HbA_1c_ test result and date, age, gender, socioeconomic status based on household income, and presence of comorbid chronic illnesses at sign-up. At 12 months, participants were again asked to report on their current HbA_1c_ level and weight.

### Statistical Analyses

Analyses were performed using SPSS, version 22.0 (IBM Corp). We examined the differences in characteristics from baseline to 12-month follow-up using paired *t* tests. The primary outcome was change in weight and HbA_1c_ level. For participants who did not report their outcomes at 12 months, we ran an intention-to-treat analysis assuming no change (ie, last observation carried forward).

An a priori power analysis using G*Power 3.1 (Heinrich Heine Universität) indicated that a total sample size of 27 would be sufficient to detect a medium effect size (*d*=0.5) with 80% power to test the difference between two dependent means using a one-tailed test and an α of .05. Thus, our proposed sample size of 45 will be more than adequate for detecting a decrease between pre- and posttest outcomes in a paired-samples *t* test.

## Results

### Participant Characteristics

At baseline, the mean HbA_1c_ level was 56.7 mmol/mol (SD 16.95; range 42.1-96.7), the mean weight was 89.4 kg (SD 13.8; range 70-135), and the mean age was 54.8 years (SD 13.2). More than half of the participants were male (26/45, 58%), 87% (39/45) were White, all were from the United Kingdom. See Table 2 for full baseline characteristics of the 45 participants.

**Table 2:** Participant characteristics at baseline.

Characteristic			Pooled (N=45)		Type 2 diabetes: HbA_1c_^a^ ≥48 mmol/mol (n=18)		Prediabetes: HbA_1c_ <48 mmol/mol (n=27)			
Age (years), mean (SD)			54.85 (13.22)		58.36 (12.46)		52.42 (13.43)			
HbA_1c_ (%), mean (SD)			7.34 (1.55)		8.86 (1.46)		6.319 (0.14)			
Weight (kg), mean (SD)			89.44 (13.81)		93.53 (17.91)		88.72 (6.69)			
**Gender, n (%)**										
	Male	26 (58)		14 (78)		12 (44)				
	Female	19 (42)		4 (22)		15 (56)				
**Ethnicity, n (%)**										
	White	39 (87)		15 (83)		24 (89)				
	Indian, Pakistani, Bangladeshi, or Arabic	5 (11)		2 (11)		3 (11)				
	Chinese, Japanese, or other East Asian	1 (2)		1 (6)		0 (0)				
**Employment, n (%)^b^**										
	Full-time employment	21 (47)		9 (50)		12 (44)				
	Part-time employment	7 (16)		1 (6)		6 (22)				
	Retired	11 (24)		5 (28)		6 (22)				
	Self-employed	4 (9)		3 (17)		1 (4)				
	Unemployed	2 (4)		0 (0)		2 (7)				

^a^HbA_1c_: hemoglobin A_1c_.

^b^Some percentages do not add up to 100 due to rounding.

### Engagement Outcomes

Of the 45 study participants recruited to the program, 37 (82%) individuals reported outcomes at 12 months, all 45 (100%) completed at least 40% of the lessons, 32 (71%) individuals completed more than nine lessons, and 29 (64%) completed all 12 core lessons of the program. Of the 45 participants, 8 (18%) did not report health outcomes but reported engagement outcomes (ie, remained engaged with the platform, which is defined as logging in within the prior 30 days).

### Retention

Of the 45 baseline participants who activated their referral, 37 (82%) reported outcomes at 12 months. For the remaining 8 people (18%) lost to follow-up, the last recorded data point was carried forward to maintain a conservative real-world evaluation.

Of the 8 people lost to follow-up, 75% (6/8) were diagnosed with prediabetes and 25% (2/8) were diagnosed with type 2 diabetes; 88% (7/8) were Caucasian and 13% (1/8) were Arab; 50% (4/8) were female; and 75% (6/8) were in full-time employment, 13% (1/8) were in part-time employment, and 13% (1/8) were retired. See Table 3 for a breakdown of characteristics.

**Table 3:** Participant characteristics of those lost to follow-up.

Characteristic		Pooled (n=8)	Type 2 diabetes: HbA_1c_^a^ ≥48 mmol/mol (n=2)	Prediabetes HbA_1c_ <48 mmol/mol (n=6)
Age (years), mean (SD)		47.3 (11.2)	49.4 (1.4)	46.6 (13.1)
HbA_1c_ (%), mean (SD)		6.57 (0.56)	7.45 (0.1)	6.28 (0.14)
Weight (kg), mean (SD)		84.76 (10.14)	90.0 (0.0)	83.02 (11.37)
**Gender, n (%)**				
	Male	4 (50)	2 (100)	2 (33)
	Female	4 (50)	0 (0)	4 (67)
**Ethnicity, n (%)^b^**				
	White	7 (88)	2 (100)	5 (83)
	Indian, Pakistani, Bangladeshi, or Arabic	1 (13)	0 (0)	1 (17)
	Chinese, Japanese, or other East Asian	0 (0)	0 (0)	0 (0)
**Employment, n (%)^b^**				
	Full-time employment	6 (75)	2 (100)	4 (67)
	Part-time employment	1 (13)	0 (0)	1 (17)
	Retired	1 (13)	0 (0)	1 (17)
	Self-employed	0 (0)	0 (0)	0 (0)
	Unemployed	0 (0)	0 (0)	0 (0)

^a^HbA_1c_: hemoglobin A_1c_.

^b^Some percentages do not add up to 100 due to rounding.

### Health Outcomes

#### HbA_1c_

Participants showed a statistically significant mean reduction in HbA_1c_ of 3.89 mmol/mol (SD 4.32; t_44_=6.03; *P*<.001). Participants who completed more than nine lessons of the program showed a larger reduction in HbA_1c_ of 4.8 mmol/mol (t_31_=5.87; *P*<.001). This is equivalent to a 7.62% mean reduction in HbA_1c_. One participant registered an HbA_1c_ increase of 6.5 mmol/mol at 12 months.

Participants with type 2 diabetes who were recruited to the Low Carb Program showed a statistically significant change in HbA_1c_ from baseline (mean 73.35 mmol/mol, SD 15.84) to 12-month follow-up (mean 67.2 mmol/mol, SD 13.59), equivalent to a mean reduction of 6.2 mmol/mol (SD 5.75; t_17_=4.56; *P*<.001). Participants who completed more than nine lessons of the program showed a statistically significant decrease in HbA_1c_ from baseline (mean 75.7 mmol/mol, SD 14.9) to 12-month follow-up (mean 68.7 mmol/mol, SD 12.8), a mean reduction in HbA_1c_ of 7.01 mmol/mol (SD 6.06; t_13_=4.33; *P*<.001). This is equivalent to an 8.81% mean reduction in HbA_1c_.

**Figure 3:**
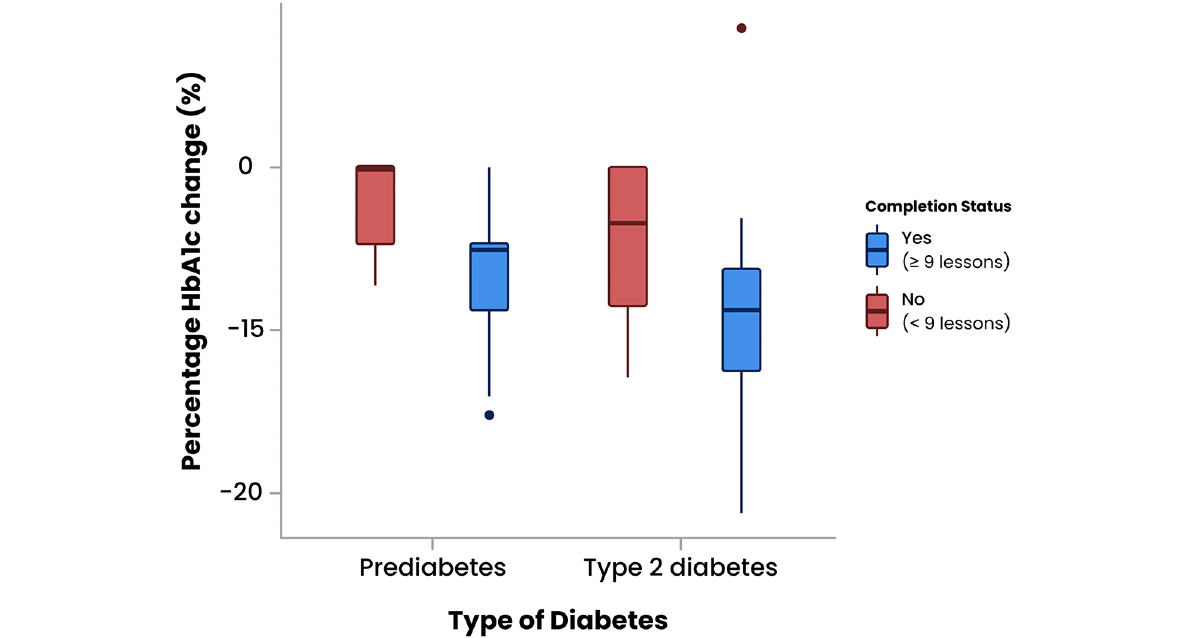
Percentage change in hemoglobin A_1c_ (HbA_1c_) from baseline to 12-month follow-up by intervention completion for prediabetes and type 2 diabetes patient groups. The boxes represent the IQRs, the lines within the boxes represent medians, and the circles represent outliers.

Participants with prediabetes who were recruited to the Low Carb Program showed a statistically significant mean reduction in HbA_1c_ of 2.35 mmol/mol (SD 1.96; t_26_=6.25; *P*<.001). Those participants who completed more than nine of the lessons did even better, reporting a mean HbA_1c_ reduction of 3.04 mmol/mol (SD 1.82) at 12 months (t_17_=7.11; *P*<.001). Results are presented in Table 4 and Figure 3.

**Table 4:** Change in HbA_1c_^a^ from baseline to 12-month follow-up by intervention completion.

Participants		Baseline HbA_1c_ (mmol/mol), mean (SD)	12-month HbA_1c_ (mmol/mol) mean (SD)	12-month HbA_1c_ change (mmol/mol), mean (SD)	12-month HbA_1c_ change (%), mean (SD)	*P* value
**Pooled (all participants)**						
	All participants (N=45)	56.68 (16.95)	52.80 (14.68)	3.89 (4.32)	6.28 (5.49)	<.001
	Completers (n=32)	58.8 (18.00)	54.0 (15.7)	4.78 (4.6)	7.62 (5.40)	<.001
	Noncompleters (n=13)	51.5 (13.23)	49.8 (12.01)	1.69 (2.53)	3.3 (4.31)	.03
**Type 2 diabetes (HbA_1c_** **≥48 mmol/mol)**						
	All participants (n=18)	73.35 (15.84)	67.20 (13.59)	6.19 (5.75)	7.96 (6.67)	<.001
	Completers (n=14)	75.7 (14.9)	68.7 (12.8)	7.01 (6.06)	8.81 (6.77)	<.001
	Noncompleters (n=4)	65.1 (18.41)	61.8 (17.10)	3.13 (0.95)	4.99 (6.22)	.18
**Prediabetes (HbA_1c_ <48 mmol/mol)**						
	All participants (n=27)	45.56 (1.49)	43.21 (2.39)	2.35 (1.96)	5.16 (4.31)	<.001
	Completers (n=18)	45.60 (1.43)	42.56 (2.38)	3.04 (1.82)	6.69 (4.01)	<.001
	Noncompleters (n=9)	45.48 (1.68)	44.51 (1.95)	0.97 (1.49)	2.11 (3.24)	.09

^a^HbA_1c_: hemoglobin A_1c_.

#### Weight

On average, participants showed a statistically significant reduction in weight, from an average of 89.44 kg (SD 13.81) at baseline to 86.67 kg (SD 13.05) at 12 months, with a mean body weight reduction of 2.77 kg (SD 2.62; t_44_=7.09; *P*<.001), equivalent to a mean total body weight reduction of 3.01% (SD 2.8). One participant registered weight gain of 1.1% body weight over the 12 months.

Participants who completed more than nine modules of the program (32/45, 71%) had an average starting weight of 91.5 kg (SD 15.12) and showed a statistically significant mean body weight reduction of 3.85 kg (SD 2.35; t_31_=9.27; *P*<.001), equivalent to a mean total body weight reduction of 4.17% (SD 2.49).

Participants with type 2 diabetes had an average starting weight of 93.53 kg (SD 17.91) that dropped to an average of 90.83 kg (SD 16.84) at 12-month follow-up, which is a statistically significant mean reduction of 2.70 kg (SD 2.21; t_17_=5.17; *P*<.001). Completers reduced their weight by an average of 3.54 kg (SD 1.7; t_17_=5.17; *P*<.001), equivalent to a mean body weight change of –3.66% (SD 2.8).

**Figure 4:**
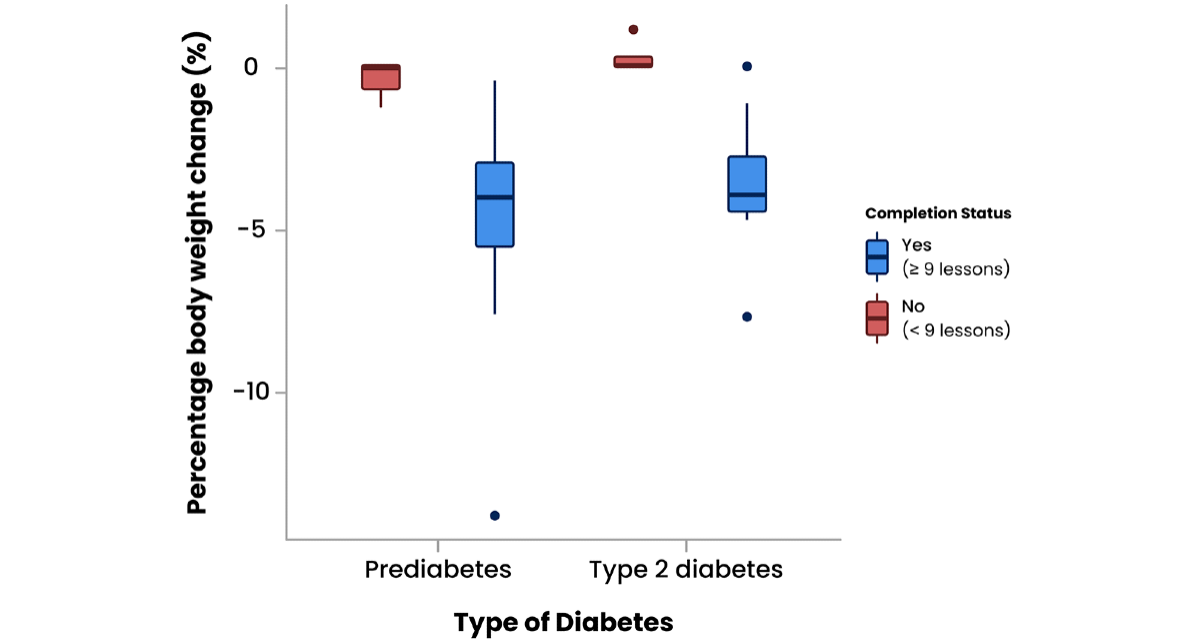
Percentage change in body weight from baseline to 12-month follow-up by intervention completion for prediabetes and type 2 diabetes patient groups. Boxes represent the IQRs, the lines within the boxes represent medians, and the circles represent outliers.

Participants with prediabetes started the program with a mean weight of 86.72 kg (SD 9.68) and reported an average weight loss of 2.82 kg (SD 2.90; t_26_=5.05; *P*<.001), equivalent to a mean body weight decrease of 3.16% (SD 3.11). Participants with prediabetes who completed more than nine lessons of the program demonstrated a greater statistically significant change in mean body weight of 4.08 kg (SD 2.77; t_17_=6.25; *P*<.001), equivalent to a mean reduction in overall body weight of 4.57% (SD 2.88). Results are presented in Table 5 and Figure 4.

**Table 5:** Change in weight from baseline to 12-month follow-up by intervention completion.

Participants		Baseline weight (kg), mean (SD)	12-month weight (kg), mean (SD)	12-month weight change (kg), mean (SD)	12-month weight change (%), mean (SD)	*P* value
**Pooled (all participants)**						
	All participants (N=45)	89.44 (13.81)	86.67 (13.05)	2.77 (2.62)	3.01 (2.80)	<.001
	Completers (n=32)	91.5 (15.12)	87.7 (14.5)	3.85 (2.35)	4.17 (2.49)	<.001
	Noncompleters (n=13)	84.4 (8.50)	84.2 (8.58)	0.12 (0.51)	0.1 (0.6)	.40
**Type 2 diabetes (HbA_1c_^a^ ≥48 mmol/mol)**						
	All participants (n=18)	93.53 (17.91)	90.83 (16.84)	2.70 (2.21)	2.78 (2.34)	<.001
	Completers (n=14)	95.5 (19.68)	92.0 (18.8)	3.54 (1.70)	3.66 (1.83)	<.001
	Noncompleters (n=4)	86.5 (7.0)	86.8 (7.18)	0.25 (0.5)	0.28 (0.5)	.39
**Prediabetes (HbA_1c_ <48 mmol/mol)**						
	All participants (n=27)	86.72 (9.68)	83.90 (9.10)	2.82 (2.90)	3.16 (3.11)	<.001
	Completers (n=18)	88.37 (9.69)	84.29 (9.25)	4.08 (2.77)	4.57 (2.88)	<.001
	Noncompleters (n=9)	83.41 (9.31)	83.12 (9.29)	0.29 (0.45)	0.34 (0.54)	.09

^a^HbA_1c_: hemoglobin A_1c_.

#### Adverse Events

There were no reported adverse events related to the intervention or that resulted in discontinuation, including no reported episodes of severe hypoglycemia.

## Discussion

### Principal Findings

This study demonstrated that signposting patients with type 2 diabetes or prediabetes to the Low Carb Program as part of routine general practice care can promote weight loss and improve glycemic control. With minimal implementation and support, this light-touch intervention was able to augment primary care workflows and demonstrated high uptake, adherence (ie, completion), and retention (ie, engagement within prior 30 days) of 45%, 64%, and 82%, respectively. There was a low dropout rate (8/45, 18%) at 12 months, which demonstrates high engagement in the platform.

For patients who completed at least nine of the program’s 12 core modules, average weight loss was 3.85 kg compared to 0.12 kg for noncompleters. The percentage of individuals who lost at least 5% of their body weight was 16% (7/45). The majority of participants registered weight loss (37/45, 82%) and 1 person gained weight (1/45, 2%). Similarly, patients who completed the program reduced their HbA_1c_ by 4.78 mmol/mol compared to 1.69 mmol/mol for those who did not complete the program. This study shows that participants who completed the intervention achieved significant weight loss and HbA_1c_ reduction at 12-month follow-up (Tables 4 and 5).

### Strengths and Limitations

This was not a randomized controlled trial, so we cannot compare the 12-month results to a control or standard-of-care group. Therefore, the results of our trial should be interpreted cautiously because this small study used convenience sampling, an open-label single-arm design, and pre-post self-reported outcomes.

However, similar to other studies on the Low Carb Program, these results support previous research that demonstrated weight loss and improved glycemic control from use of the intervention in adults diagnosed with type 2 diabetes and prediabetes [25,31,32]. This study showed high engagement and retention. Evidence suggests that digital solutions that are personalized to the user and have clinical endorsement may positively affect participant engagement [33,34]. Repeated in a larger practice, this research could have a significant impact.

One of the limitations of the research was that patients who were offered access to the Low Carb Program but chose not to accept it or did not complete the sign-up were not tracked or followed up. Although beyond the scope of this study, further research should explore reasons for signposting refusal. Another limitation was the self-reported nature of the data, as only data from Bluetooth-enabled weighing scales and self-input were collected. However, it has been shown that self-reported health outcomes are similar to actual values [35,36]. Although beyond the scope of this feasibility study, future research should extract HbA_1c_, weight, and medication data directly from general practice record systems rather than rely on patients’ self-report of this data.

Those who completed the program lost more weight than those who did not. Previous research has shown that participant motivation affects continuing intervention adherence and, as such, introduces a self-selection bias to the data, as participants who continue to adhere are more likely to have lost weight [37]. Another limitation is lack of specific feature usage data. Without feature usage data, investigating differences in outcomes or engagement with specific features of the program was not possible.

### Comparison With Prior Work

Findings from this study are comparable to other similar interventions. The platform has been shown to have a high engagement rate and to be noninferior to other in-person or online interventions [38-40]. Given the brief intervention that was provided, we were able to achieve high uptake within the context of general practice and primary care. Typically, other interventions require staff resources and time.

HeLP-Diabetes (Healthy Living for People with type 2 Diabetes), an online type 2 diabetes self-management tool, reported a lower uptake, engagement, and completion rate. HeLP-Diabetes required staff to identify eligible patients and recruited patients through consultations and text messages [41]. A total of 23% of patients engaged past the first HeLP-Diabetes module, and 9.4% of patients completed the program.

The program mirrors outcomes for intensive diabetes interventions, such as Virta, which combines coaching and teleconsultation and reported an 83.2% retention rate at 1 year [42]. Noom, a diabetes prevention program in the United States, showed a similar engagement rate of 77.6% at 1 year [43].

A large nonrandomized trial of an online diabetes prevention program that provided digital education, a live e-coach, and virtual groups in North America showed similar weight loss at 12 months (4.0 kg) [44] to our study. GlycoLeap, a Singaporean mobile lifestyle management program reported a mean weight loss of 2.0 kg at 12-week follow-up (mean –2.0 kg, SD 1.6; *P*<.001) [45]. An evaluation of Time2Focus, a self-guided app for diabetes education in North America, showed a lower mean HbA_1c_ reduction at follow-up (mean –0.39 mmol/mol; β=.06; *P*=.78) [46].

This research suggests that similar to digital interventions such as HeLP-Diabetes, the mode of delivery is acceptable to both providers and patients [47]. With the growing burden of type 2 diabetes, prescribing digital health interventions to encourage behavior change can enable health care providers to support patients remotely, at scale, and enables health care providers to focus on high-risk or high-priority patients.

### Conclusions

The majority of participants who registered for the intervention lost weight and improved glycemic control. Although our study design does not support causal conclusions, this real-world evaluation suggests that the intervention can be a useful adjunct for lifestyle self-management for adults with type 2 diabetes and prediabetes. Further research should explore the impact on larger groups of patients, explore the acceptability of intervention features, and refine engagement strategies to maximize uptake, completion rates, and patient outcomes.

## Appendix

Multimedia Appendix 1 Screenshots of the Low Carb Program multi-platform app on the web, a mobile phone, a smart watch, and a smart speaker.

Multimedia Appendix 2 Examples of personalized patient journeys in the Low Carb Program.

## Abbreviations

COM-B: Capability-Opportunity-Motivation Behavior
CONSORT: Consolidated Standards of Reporting Trials
HbA_1c_: hemoglobin A_1c_
HeLP-Diabetes: Healthy Living for People with type 2 Diabetes
NHS: National Health Service
NICE: National Institute for Health and Care Excellence
