# Supplementary figures and images for: Evaluation of the Low Carb Program Digital Intervention for the Self-Management of Type 2 Diabetes and Prediabetes in an NHS England General Practice: Single-Arm Prospective Study
Source: JMIR Diabetes. 2021 Sep 9;6(3):e25751. doi: 10.2196/25751 (PMC8461529; doi:10.2196/25751)

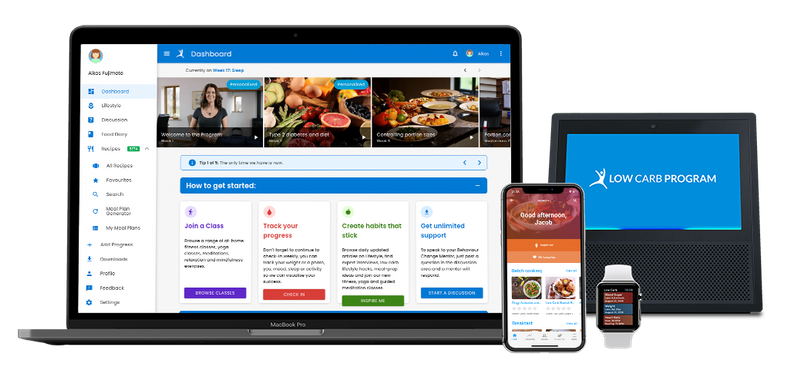

Supplement: Multimedia Appendix 1 [file diabetes_v6i3e25751_app1.png]
