# Supplementary material for: Evaluation of the Low Carb Program Digital Intervention for the Self-Management of Type 2 Diabetes and Prediabetes in an NHS England General Practice: Single-Arm Prospective Study
Source: JMIR Diabetes. 2021 Sep 9;6(3):e25751. doi: 10.2196/25751 (PMC8461529; doi:10.2196/25751)
Supplement: Multimedia Appendix 2 [file diabetes_v6i3e25751_app2.pdf]

Appendix 2: Patient case examples

The following patient case studies demonstrate the method in which the Low Carb Program personalises the patient experience to baseline data characteristics.

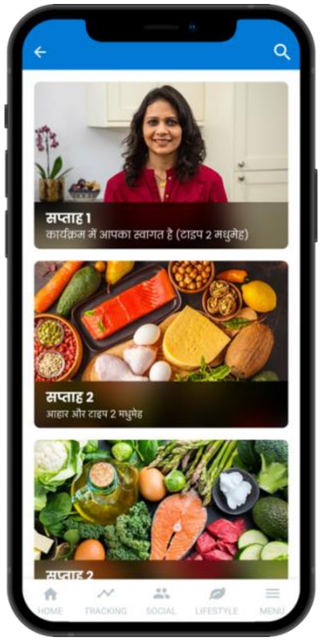

Patient A

Patient A is a 60 year old South Asian female diagnosed with type 2 diabetes. Patient A speaks Hindi as her first language and is a vegetarian. On signing up to the program, Patient A's registration data personalises her experience of the Low Carb Program. Patient A is presented with structured education that explains the physiology of type 2 diabetes and how to self-manage type 2 diabetes through reducing carbohydrates in the diet. Patient A's experience is provided in Hindi (native-language) delivering information to meet cultural needs and expectations. Patient A is supported with vegetarian meal plans that are provided in Punjabi and ingredients that match cultural expectations. Virtual meetups that Patient A engages in are facilitated by a Hindi-speaking coach should she choose to engage with this component of the Low Carb Program.

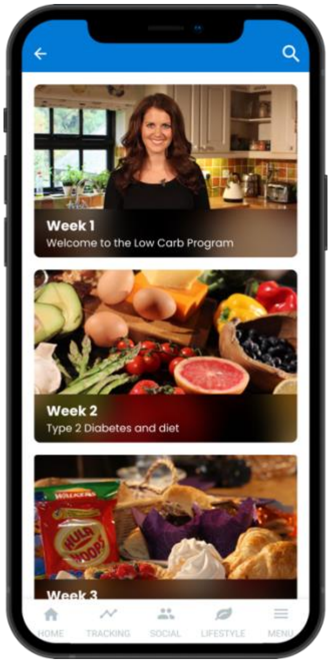

Patient B

Patient B is a 52 year old Caucasian male diagnosed with prediabetes. Patient B speaks English as his first language, is a smoker, eats a standard diet and is allergic to dairy. On signing up to the program, Patient B's registration data personalises his experience of the Low Carb Program. Patient B is presented with structured education that explains the physiology of prediabetes and how to self-manage prediabetes through reducing carbohydrates in the diet. Patient B's experience is provided in English (native-language). Patient B is supported with meal plans that exclude dairy and are provided in English. Virtual meetups that Patient B engages in are facilitated by an English-speaking coach should he choose to engage with this component of the Low Carb Program.
